# Supplementary material for: Augmented CPT1A Expression Is Associated with Proliferation and Colony Formation during Barrett’s Tumorigenesis
Source: Int J Mol Sci. 2022 Oct 4;23(19):11745. doi: 10.3390/ijms231911745 (PMC9570428; doi:10.3390/ijms231911745)
Supplement: Supplementary file 1 [file ijms-23-11745-s001.zip › ijms-1910376-supplementary.pdf]

Supplementary Files

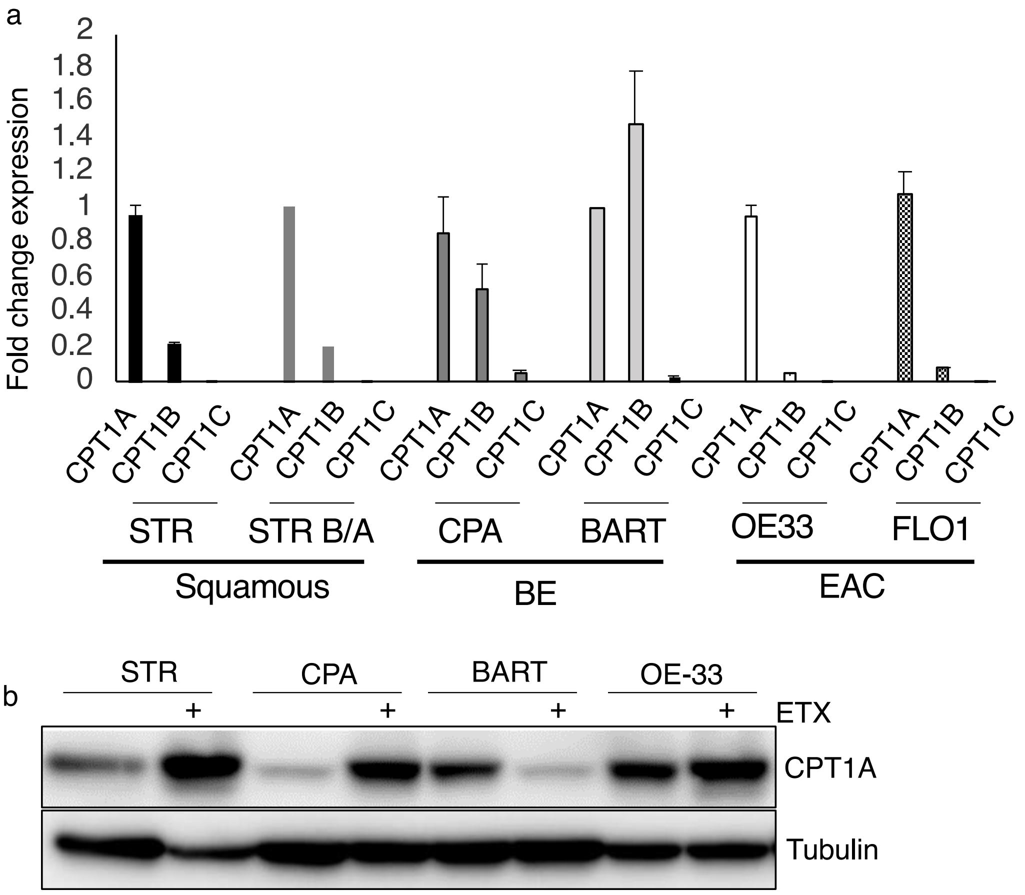

Figure S1: *CPT1A* is the main *CPT1* gene expressed in esophageal cells lines and induced after upon functional inhibition with etomoxir in most cell lines tested. (a) qRT-PCR using primers for RNA amplification of *CPT1A, CPT1B* and *CPT1C* shows that *CPT1A* is dominantly expressed in all esophageal cell lines except BAR-T, on which *CPT1B* is expressed at a similar level. (b) Protein extracts probed for CPT1A using Western Blot shows that 10 μM etomoxir (ETX) induce protein expression in most cell lines except BAR-T.

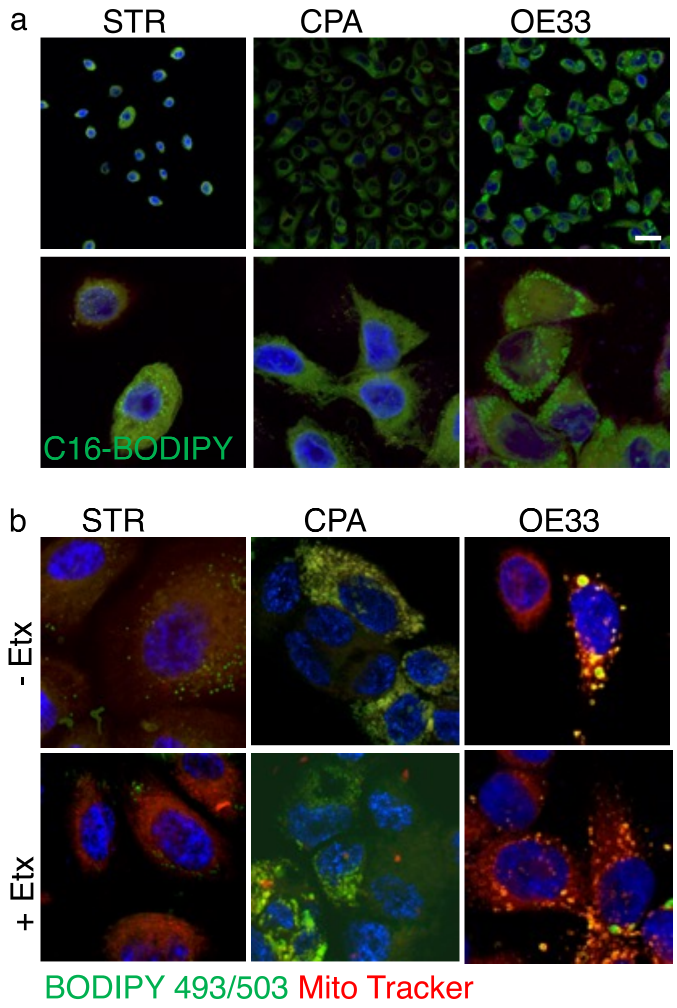

Figure S2: Lipid uptake and accumulation upon inhibition of CPT1A in esophageal cells. (a) C16-BODIPY is taken up by all esophageal cell lines tested and accumulates in focal vesicles or lipid droplets as visible by the green fluorescence signal. (b) Labeling of mitochondria using MitoTracker (red) and lipids using BODIPY 493/503 (green) demonstrates the co-localization of BODIPY-labeled neutral lipids and mitochondria, especially in OE44 cells (yellow). Upon inhibition of CPT1A with etomoxir (Etx), less co-localization is observed as measured by a lower yellow signal. Representative images shown.

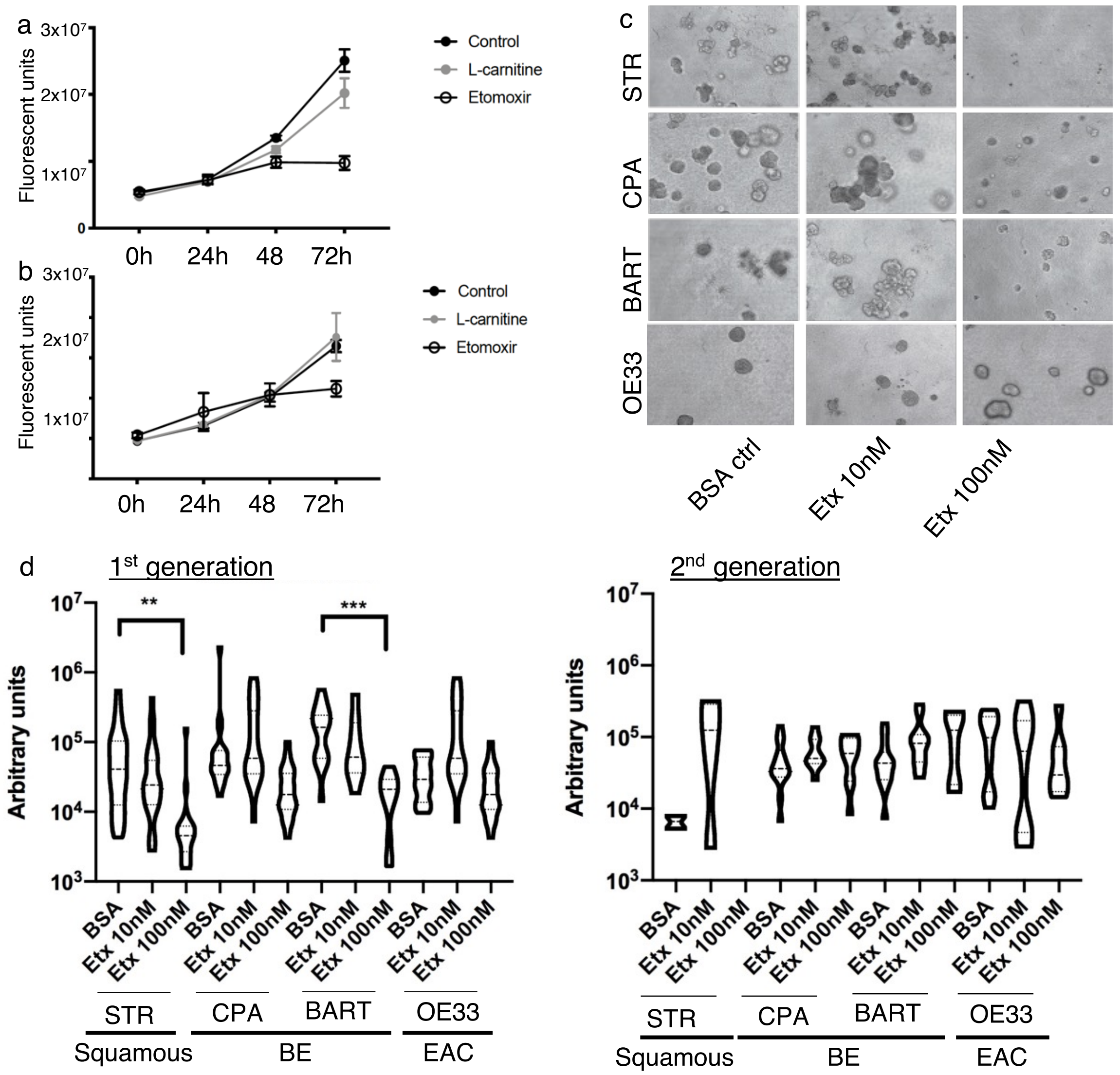

Figure S3: Inhibition of FAO using etomoxir inhibits proliferation and decreased spheroid size dose-dependently. Using the CPT1A inhibitor etomoxir (Etx), proliferation is decreased in (a) and (b) cells when compared to conditions with L-carnitine mediated FAO stimulation. (c) STR, CPA, BAR-T and OE33 were grown as spheroids in 2% Matrigel and treated with 10nM and 100nM etomoxir (Etx). The higher concentration inhibited spheroid growth in normal and BE cell lines but not in the EAC cell line OE33. (d) Quantification of spheroid size in the presence of Etx shows a dose-dependent decrease for 1st generation spheroids, significant for STR and BAR-T cells. When spheroids were dispersed and seeded for 2nd generation spheroid growth normal STR cells formed limited size spheroids compared to BE and EAC cells and no spheroids in the presence of high concentration Etx while secondary spheroids for all other cell lines were unaffected by Etx. *$p < 0.05$, **$p < 0.01$, ***$p < 0.001$, ****$p < 0.0001$, One-way ANOVA.

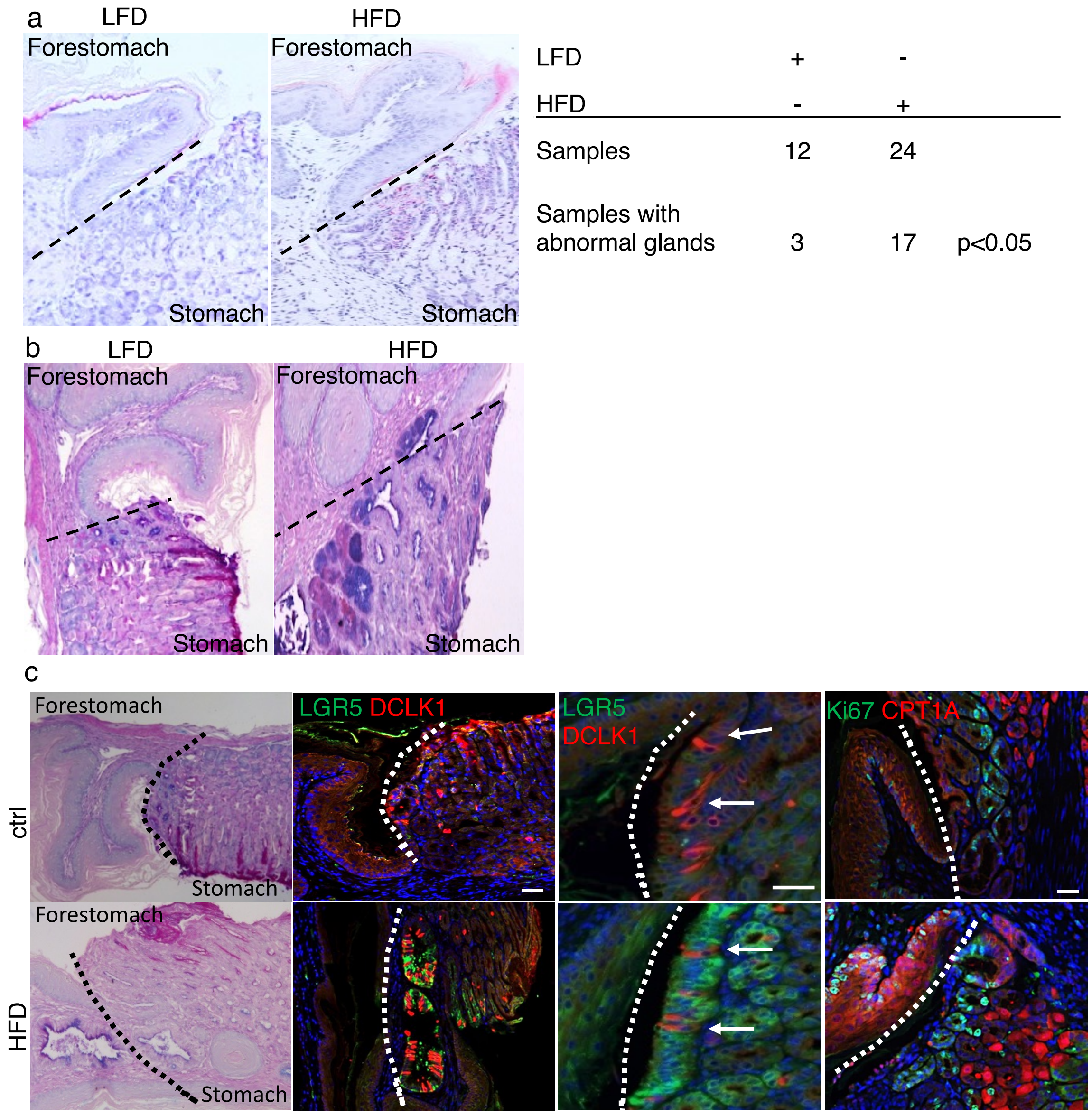

LFD	+	-	
HFD	-	+	
Samples	12	24	
Samples with abnormal glands	3	17	$p<0.05$

Figure S4: High fat diet (HFD) in C57/B6 mice induces CPT1A expression at the squamocolumnar junction. (a) Hematoxylin/Eosin staining show changes comparable with epithelial metaplasia at the junction between forestomach and stomach (dashed line) and abnormally enlarged glands. (b) Alcian Blue/PAS staining was used to detect the expression of mucins in the metaplastic areas and identify mucin-positive cells in the enlarged glands at the squamocolumnar junction in HFD-fed mice (dashed line). (c) Using immunofluorescence staining, we detected increased LGR5+ and DCLK1+ stem cell marker expression in the metaplastic areas of HFD-mice. A magnification of the DCLK1-positive cells (white arrows) shows a Tuft cell-like morphology. Ki67 and CPT1A were also increased in the HFD-mice compared to LFD control mice. Scale bar, 50 micron.

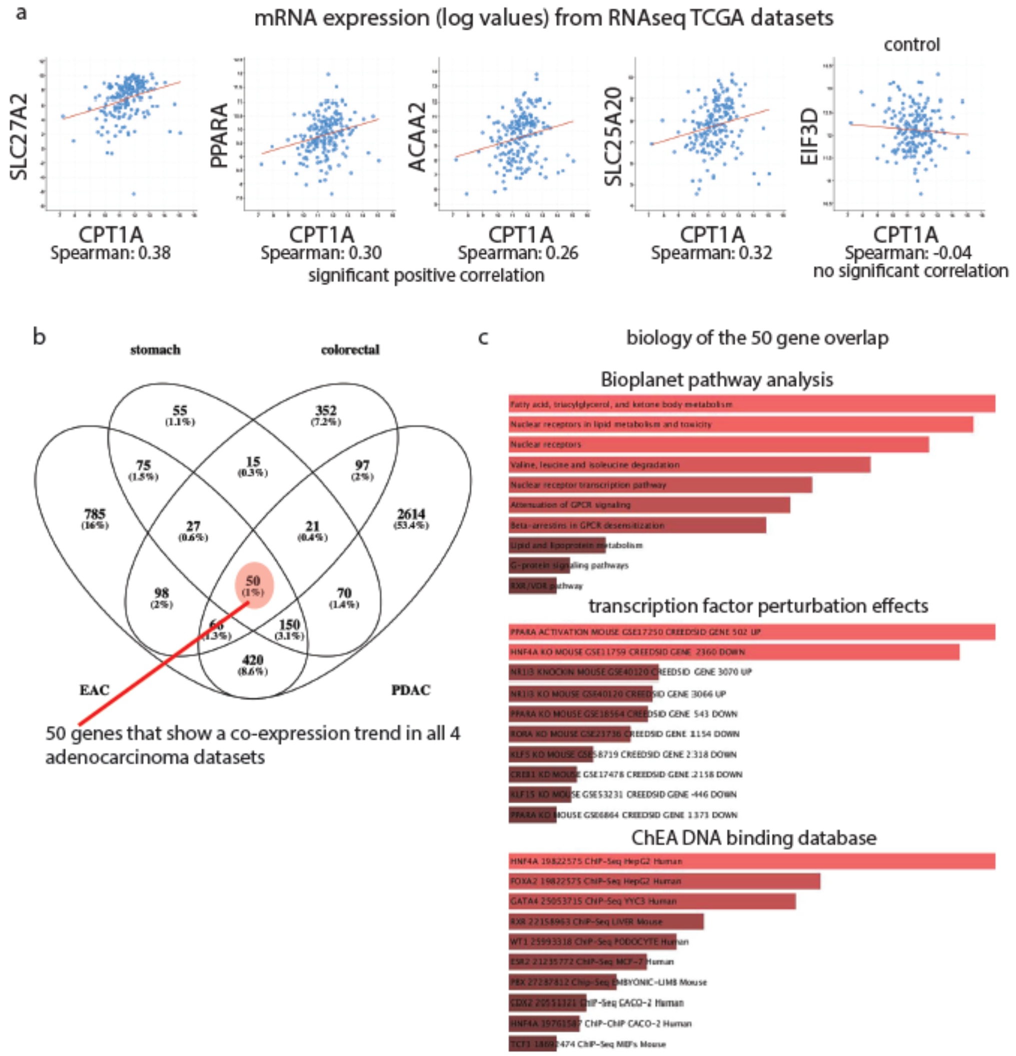

Figure S5: Association of CPT1A expression with fatty acid oxidation in several adenocarcinoma datasets of the digestive tract (EAC, stomach, colorectal, pancreas). (a) Scatter plots to demonstrate co-gene expression of CPT1A with fatty acid oxidation related (SLC27A2 y=0.63x+ -0.4, R^2= 0.08; PPARA y=0.16x+7.96, R^2= 0.07; ACAA2 y=0.31x+6.01, R^2= 0.05, SLC25A20 y=0.21x+5.3, R^2= 0.04) genes in EAC samples, which reach a Spearman correlation factor of >0.2. (b) A comparison of genes co-expressed with CPT1A in digestive tract adenocarcinomas results in the core set of 50 genes. (c) These 50 genes show an association with PPARA, HNF4A, CDX2 and FOXA2 transcription factor binding in their respective promoter regions and pathways terms related to fatty acid biology. For co-expression analysis we used the cbio database (https://www.cbioportal.org) and selected datasets related to adenocarcinomas of the digestive tract. Co-expressed genes with CPT1A were selected with a cut-off of a Spearman's correlation factor >0.2. The resulting gene set was fed into ENRICHR (https://maayanlab.cloud/Enrichr/) for further analysis.
